# Influence of Statistics on Quackery

**Published:** 1848

**Authors:** 


### ARTICLE IV.

### *Influence of Statistics on Quackery.*

Dr. Cartwright, of Natchez, Miss., delivered an excellent lecture recently in St. Louis, which has been published in the Missouri Medical and Surgical Journal, from which we make the following extract, showing most clearly the influence of empiricism on the public mortality. If such facts as these were carefully collected wherever opportunities offer, and given to the public, the influence could scarcely be different from that on the community of Natchez:

Permit me, gentlemen, now to make some remarks on the results afforded by Statistical Medicine, in the different eras above mentioned. In the first era, extending from 1823 to 1834—ten years—empiricism was excluded from Mississippi by the strong hand of legislative power. During that period, the annual average mortality of the whole population in the city of Natchez, including strangers, boatmen, and great numbers of negroes brought there for sale, was only one in thirty, and not more than one in sixty of the citizens proper. Yet these ten years constituted a period rife with epidemic diseases. During this period, besides the diseases incident to the climate, the physicians had to contend with the small-pox, measles, whooping-cough, scarlet-fever, and Asiatic cholera, all in the epidemic form, and besides, with two epidemic yellow-fevers. But they had no quackery to contend with. They had the confidence of the people. The venders, proprietors, and pedlars, in patent medicines and nostrums, had not destroyed that confidence, as they have since done, to open a market for the sale of their empirical preparations. The citizens of Natchez, leaning on the arm of science, under circumstances calculated to desolate and depopulate any place less fortunately supplied with skilful physicians, passed through a period of ten years, and actually lost a fewer number per annum, in proportion to population, than almost any other town or city from which we have any authentic records—the mortality among the citizens proper, not exceeding one in sixty, while the whole mortality, including strangers, was only one in thirty. The sum total of all the deaths per annum, occurring both among the resident and non-resident population, during the whole period of ten years indicates a far less annual average mortality than either Edinburgh, Dublin, Madrid, Amsterdam, Naples, Rome, Vienna, or France itself can boast of. This result may be accounted for, from the fact, that the citizens generally of Natchez, sought medical aid very early in their disease, and all had the benefit of regular medical advice; whereas in France, and the cities just mentioned, only a small portion of the population, comparatively, have the advantages of regular medical attendance, and the necessary accompaniaments of a well regulated sick room from the commencement of their disease.

Let us now come to the second era, when the laws forbidding the empirical practice of medicine in Mississippi had been annulled under the new constitution, viz.: early in the year 1834. No less than six empirics, under the name of reformers, soon afterwards, located themselves in Natchez. They endeavored to convince the people that the regular physicians were unworthy of their confidence; that the old fashioned ignorance of the regular physicians, (or mineral medicine doctors as they called them,) ignorance disguised by technical terms in Latin and Greek, caused much unnecessary mortality, and greatly increased the amount of human suffering; whereas, they (the reformers) had a new system of treating all diseases, much safer, better, and *cheaper*, than the old method. They entered on the work of reformation, and the sexton's book proves, that no sooner did they enter on their work, than the mortality suddenly increased from one in thirty to one in twenty-one per annum, almost equal to the mortality among the free negroes in Philadelphia. In a time remarkable for its health, without any epidemic disease to contend with, and omitting entirely every death which occurred in the epidemic yellow-fever of 1837, Natchez, during a period of four years and six months, (omitting the three months of the epidemic,) lost sixty-two individuals over and above the sum total of all the deaths in the ten years preceeding the advent of the reformers. It is true, the popluation in the second period was somewhat greater than that of the first period, but by no means great enough to account for the increase of mortality. The exact population being known, and the number of deaths being also known, the evils of empiricism can be demonstrated with certainty, as likewise the beneficial influences of science in medicine. Thus, in 1830, the exact population was known from a census carefully taken that year. The mortality of that year, when the people were protected from empiricism, was only one in thirty-four. The annual ratio of mortality in 1837, when the population was known, from an accurate census of the inhabitants taken that year, was one in twenty-four, for the nine healthy months, omitting entirely the three months of the epidemic. During the whole period of the reign of empiricism, from 1834 to near the termination of 1839, the annual average mortality, making more than due allowance for the increase of population, was about one in twenty-one per annum; whereas, before the introduction of quackery it was only one in thirty for ten years in succession. Lest it might be supposed that the comparatively small mortality of the ten years of the first period, when empiricism was excluded, might be owing to a greater proportional number of citizens leaving town in the summer, and likewise to a smaller number of unacclimated persons, it will be found on examination, that the mortality in January, February, March, April, and May, for five years in succession, has been nearly twice as great, under empiricism (making every allowance for increase of population) as it had been in the corresponding months for ten years under the regular practice. I do not attribute the whole of the increased mortality of the second period to the direct agency of the empirics themselves. The greater part of it was doubtlessly owing to their indirect agency in making war against some of the best known and well tried remedies of the Materia Medica, and to the artful measures they took, in conjunction with the proprietors and venders of nostrums, to impair the confidence of the public in the regular medical profession. Distrust and want of confidence in physicians will always increase the mortality among any people, by preventing timely application for assistance. Statistical Medicine, as gathered from the sexton's books of Natchez, proves that a large number of the deaths occurring during the reign of empiricism, were not reported by any practitioner whatever. The people being told that there were two systems in medicine, an old system full of errors and dangers, and a new or reformed system, neglected, in many cases, to avail themselves of either, being doubtful which to choose.

About the close of 1839, after nearly five years had elapsed under the reign of quackery, called a reformation in medicine, I commenced a numerical analysis to test the results of the empirical practice. When completed, I called the physicians together and showed them the result. The numerical method declared that the mortality had nearly doubled under the pretended reformation in medicine; that is, it was nearly twice as great since the empirics came to town and opened their shops, as it had been during any portion of the preceeding time when medicine was protected. I called for a committee to examine my data, to compare them with the sexton's book and the different censes of the population, and to see if any error had been made, either in the statement of facts or the deductions therefrom, and to confer with the city council and the health officer, as also with the other officers of the city in regard to the actual number of the population. The committee reported that the facts were true, and the deductions founded thereon were mathematically correct; that it was strictly true that the mortality, from the very first appearance of the reformers in Natchez, had nearly doubled the mortality previously—in a healthy time with no epidemics to contend with; that the deaths occurring in the only epidemic which occurred during the reformation, had not been taken in the calculations at all, while all the deaths occurring in two epidemic yellow-fevers, two epidemic choleras, and one epidemic scarlet-fever, measles, whooping-cough, and small-pox, which afflicted Natchez previously to the advent of the reformers, had been included in the mortality of that period when medicine was solely in the hands of the regular physicians. Yet the mortality of this period, so rife with epidemics, was scarcely half as great as the mortality of that period when quackery was the only epidemic which prevailed. The prevalence of quackery, therefore, had been more fatal to the citizens of Natchez, nearly two to one, than the prevalence of the yellow-fever, cholera, measles, whooping-cough, and small-pox, and all other diseases put together. These facts were embodied in a discourse before the Natchez Medical Society, and Dr. Yandell procured a copy from me, and published it in the Louisville Medical Journal. No other journal took notice of it, and no other publication was made of it, physicians being averse to using the public press. Nevertheless the citizens of Natchez found out that the result of introducing quackery had done no other good than nearly to double the mortality, and had made Natchez in a healthy time more fatal to human life than it ever had been before the introduction of empiricism in the most sickly times. They therefore petitioned the legislature to exclude it. As soon as the empirics found that the truth was against them, and that instead of their practice curing everything, it had been about twice as fatal as the old practice, and that the citizens had begun to find it out, they began, one by one, to exclude themselves — that is, to back out, and to leave the field to the physicians again. Their practice rapidly declined, and that of the physicians increased by the end of the year 1839. From that time the third era commenced, when quackery began to die a natural death under the light of statistical medicine, and soon became almost totally extinguished. As quackery declined, the mortality diminished, and Natchez became once more a healthy city, as the sexton's books will prove. In the summer of 1847, however, after the old fashioned, undisguised quackery had died out, one of the old physicians, who had gained a high reputation from a liberal and early use of quinine in malarial diseases, introduced empiricism again in Natchez, dressed in the disguise of science, under the new appellation of Homœopathy. From the summer, therefore, of 1847, the fourth era begins. Sufficient time has not yet elapsed to test the results of the practice by the unerring results of Statistical Medicine.

